# 4-Hydr­oxy-*N*′-(4-hydroxy­benzo­yl)benzo­hydrazide

**DOI:** 10.1107/S1600536809012136

**Published:** 2009-04-08

**Authors:** Kong Mun Lo, Seik Weng Ng

**Affiliations:** a Department of Chemistry, University of Malaya, 50603 Kuala Lumpur, Malaysia

## Abstract

In the mol­ecule of the title compound, C_14_H_12_N_2_O_4_, the two benzene rings make a dihedral angle of 84.53 (8)°. O—H⋯O and N—H⋯O hydrogen bonds link adjacent mol­ecules into a layer structure.

## Related literature

For the unsubstituted parent compound, 1,2-dibenzoyl­hydrazine, see: Shanmuga Sundara Raj *et al.* (2000 ▶). For the 2-hydroxy ­substituted compound, 1,2-disalicyloylhydrazine, see: Chen *et al.* (2008 ▶).
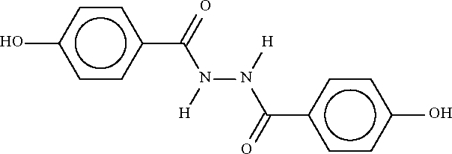


## Experimental

### 

#### Crystal data

C_14_H_12_N_2_O_4_
*M* *_r_* = 272.26
Orthorhombic, 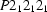

*a* = 8.7058 (7) Å
*b* = 9.7646 (8) Å
*c* = 14.258 (1) Å
*V* = 1212.05 (16) Å^3^
*Z* = 4
Mo *K*α radiation
μ = 0.11 mm^−1^
*T* = 123 K
0.40 × 0.15 × 0.10 mm

#### Data collection

Bruker SMART APEX diffractometer
Absorption correction: none
6928 measured reflections
1599 independent reflections
1475 reflections with *I* > 2σ(*I*)
*R*_int_ = 0.021

#### Refinement

*R*[*F* ^2^ > 2σ(*F* ^2^)] = 0.030
*wR*(*F* ^2^) = 0.088
*S* = 1.04
1599 reflections
197 parameters
4 restraints
H atoms treated by a mixture of independent and constrained refinement
Δρ_max_ = 0.27 e Å^−3^
Δρ_min_ = −0.17 e Å^−3^

### 

Data collection: *APEX2* (Bruker, 2008 ▶); cell refinement: *SAINT* (Bruker, 2008 ▶); data reduction: *SAINT*; program(s) used to solve structure: *SHELXS97* (Sheldrick, 2008 ▶); program(s) used to refine structure: *SHELXL97* (Sheldrick, 2008 ▶); molecular graphics: *X-SEED* (Barbour, 2001 ▶); software used to prepare material for publication: *publCIF* (Westrip, 2009 ▶).

## Supplementary Material

Crystal structure: contains datablocks global, I. DOI: 10.1107/S1600536809012136/xu2507sup1.cif

Structure factors: contains datablocks I. DOI: 10.1107/S1600536809012136/xu2507Isup2.hkl

Additional supplementary materials:  crystallographic information; 3D view; checkCIF report

## Figures and Tables

**Table 1:** Hydrogen-bond geometry (Å, °)

*D*—H⋯*A*	*D*—H	H⋯*A*	*D*⋯*A*	*D*—H⋯*A*
O1—H1o⋯O2^i^	0.84 (1)	1.85 (1)	2.684 (2)	172 (3)
O4—H4o⋯O3^ii^	0.85 (1)	1.83 (1)	2.675 (2)	178 (3)
N1—H1n⋯O2^iii^	0.88 (1)	2.08 (1)	2.920 (2)	162 (2)

Symmetry codes: (i) 
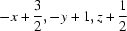
; (ii) 
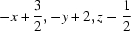
; (iii) 
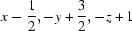
.

**Fig. 1.**
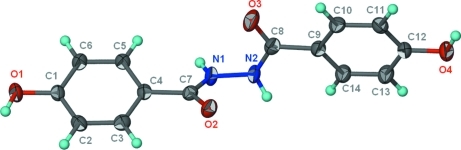
Thermal ellipsoid plot (Barbour, 2001) of C14H12N2O4 at the 70% probability level. Hydrogen atoms are drawn as spheres of arbitrary radius.

## supplementary crystallographic information

### Experimental

4-Hydroxybenzoylhydrazine (0.31 g, 2 mmol) and pyruvic acid (0.16 g, 2 mmol) were heated in ethanol (100 ml) for 3 h in an attemp to synthesize a Schiff base. Slow cooling of the filtered solution gave crystals of the hydrazide.

### Refinement

Carbon-bound H-atoms were placed in calculated positions (C–H 0.95 Å) and were included in the refinement in the riding model approximation, with *U*(H) set to 1.2*U*(C). The amino and hydroxy H-atoms were located in a difference Fourier map, and were refined with distance restraints of O–H 0.84±0.01 Å and N–H 0.88±0.01 Å. Their temperature factors were refined.

Some 1159 Friedel pairs were merged.

### Crystal data

**Table:**

C_14_H_12_N_2_O_4_	*F*(000) = 568
*M**_r_* = 272.26	*D*_x_ = 1.492 Mg m^−^^3^
Orthorhombic, *P*2_1_2_1_2_1_	Mo *K*α radiation, λ = 0.71073 Å
Hall symbol: P 2ac 2ab	Cell parameters from 3150 reflections
*a* = 8.7058 (7) Å	θ = 2.7–28.1°
*b* = 9.7646 (8) Å	µ = 0.11 mm^−^^1^
*c* = 14.258 (1) Å	*T* = 123 K
*V* = 1212.05 (16) Å^3^	Prism, light yellow
*Z* = 4	0.40 × 0.15 × 0.10 mm

### Data collection

**Table:**

Bruker SMART APEX diffractometer	1475 reflections with *I* > 2σ(*I*)
Radiation source: fine-focus sealed tube	*R*_int_ = 0.021
graphite	θ_max_ = 27.5°, θ_min_ = 2.5°
ω scans	*h* = −7→11
6928 measured reflections	*k* = −12→12
1599 independent reflections	*l* = −18→18

### Refinement

**Table:**

Refinement on *F*^2^	Primary atom site location: structure-invariant direct methods
Least-squares matrix: full	Secondary atom site location: difference Fourier map
*R*[*F*^2^ > 2σ(*F*^2^)] = 0.030	Hydrogen site location: inferred from neighbouring sites
*wR*(*F*^2^) = 0.088	H atoms treated by a mixture of independent and constrained refinement
*S* = 1.04	*w* = 1/[σ^2^(*F*_o_^2^) + (0.0567*P*)^2^ + 0.1781*P*] where *P* = (*F*_o_^2^ + 2*F*_c_^2^)/3
1599 reflections	(Δ/σ)_max_ = 0.001
197 parameters	Δρ_max_ = 0.27 e Å^−^^3^
4 restraints	Δρ_min_ = −0.17 e Å^−^^3^

### Fractional atomic coordinates and isotropic or equivalent isotropic displacement parameters (Å^2^)

**Table:**

	*x*	*y*	*z*	*U*_iso_*/*U*_eq_	
O1	0.60946 (17)	0.59285 (14)	0.90517 (8)	0.0287 (3)	
O2	0.72656 (15)	0.62247 (13)	0.46567 (8)	0.0247 (3)	
O3	0.65093 (19)	0.92446 (15)	0.39648 (9)	0.0364 (4)	
O4	0.63007 (17)	0.90074 (14)	−0.04878 (8)	0.0310 (3)	
N1	0.49389 (17)	0.72003 (15)	0.47376 (9)	0.0217 (3)	
N2	0.49553 (19)	0.74438 (15)	0.37802 (9)	0.0223 (3)	
C1	0.6163 (2)	0.60858 (17)	0.81114 (11)	0.0206 (3)	
C2	0.7010 (2)	0.52136 (18)	0.75329 (12)	0.0224 (4)	
H2	0.7591	0.4487	0.7799	0.027*	
C3	0.7002 (2)	0.54091 (17)	0.65708 (12)	0.0222 (4)	
H3	0.7585	0.4818	0.6179	0.027*	
C4	0.61465 (19)	0.64672 (17)	0.61727 (11)	0.0191 (3)	
C5	0.5318 (2)	0.73400 (18)	0.67564 (11)	0.0214 (3)	
H5	0.4735	0.8065	0.6491	0.026*	
C6	0.5334 (2)	0.71626 (18)	0.77205 (11)	0.0235 (4)	
H6	0.4781	0.7774	0.8114	0.028*	
C7	0.6172 (2)	0.66161 (17)	0.51417 (11)	0.0197 (3)	
C8	0.5846 (2)	0.84605 (18)	0.34327 (12)	0.0226 (4)	
C9	0.5944 (2)	0.85689 (17)	0.23956 (12)	0.0210 (4)	
C10	0.7023 (2)	0.94658 (17)	0.20197 (12)	0.0224 (4)	
H10	0.7664	0.9979	0.2429	0.027*	
C11	0.7180 (2)	0.96226 (18)	0.10568 (12)	0.0233 (4)	
H11	0.7928	1.0232	0.0809	0.028*	
C12	0.6230 (2)	0.88771 (17)	0.04537 (11)	0.0222 (4)	
C13	0.5156 (2)	0.79694 (19)	0.08191 (11)	0.0265 (4)	
H13	0.4517	0.7455	0.0409	0.032*	
C14	0.5017 (2)	0.78146 (18)	0.17811 (12)	0.0247 (4)	
H14	0.4285	0.7189	0.2027	0.030*	
H1O	0.668 (3)	0.529 (2)	0.9222 (17)	0.045 (7)*	
H4O	0.698 (2)	0.958 (2)	−0.0659 (18)	0.050 (8)*	
H1N	0.4167 (19)	0.757 (2)	0.5039 (15)	0.031 (6)*	
H2N	0.467 (3)	0.6748 (17)	0.3437 (14)	0.046 (7)*	

### Atomic displacement parameters (Å^2^)

**Table:**

	*U*^11^	*U*^22^	*U*^33^	*U*^12^	*U*^13^	*U*^23^
O1	0.0391 (8)	0.0299 (7)	0.0172 (6)	0.0076 (6)	−0.0011 (5)	0.0049 (5)
O2	0.0253 (6)	0.0290 (6)	0.0199 (5)	0.0028 (6)	0.0036 (5)	−0.0039 (5)
O3	0.0500 (9)	0.0399 (8)	0.0194 (6)	−0.0174 (7)	−0.0003 (6)	−0.0066 (6)
O4	0.0440 (9)	0.0313 (7)	0.0179 (6)	−0.0098 (7)	0.0005 (6)	0.0030 (5)
N1	0.0219 (7)	0.0300 (7)	0.0132 (6)	0.0027 (7)	0.0014 (6)	−0.0001 (6)
N2	0.0265 (8)	0.0276 (7)	0.0128 (6)	−0.0014 (7)	−0.0012 (6)	−0.0004 (5)
C1	0.0244 (9)	0.0209 (7)	0.0167 (7)	−0.0032 (7)	−0.0023 (6)	0.0014 (6)
C2	0.0238 (9)	0.0207 (7)	0.0227 (8)	0.0008 (7)	−0.0022 (7)	0.0036 (6)
C3	0.0216 (9)	0.0221 (8)	0.0230 (8)	0.0012 (7)	0.0014 (7)	−0.0016 (6)
C4	0.0192 (8)	0.0208 (7)	0.0173 (7)	−0.0039 (7)	−0.0004 (6)	0.0002 (6)
C5	0.0236 (9)	0.0206 (7)	0.0199 (7)	0.0021 (7)	−0.0005 (7)	0.0008 (6)
C6	0.0283 (9)	0.0236 (8)	0.0186 (8)	0.0026 (8)	0.0019 (7)	−0.0018 (7)
C7	0.0208 (8)	0.0188 (7)	0.0194 (8)	−0.0029 (7)	0.0009 (6)	−0.0025 (6)
C8	0.0231 (9)	0.0247 (8)	0.0201 (8)	0.0015 (7)	0.0006 (7)	−0.0026 (7)
C9	0.0231 (8)	0.0217 (8)	0.0181 (8)	0.0019 (7)	−0.0001 (6)	−0.0009 (6)
C10	0.0230 (9)	0.0217 (8)	0.0224 (8)	−0.0009 (7)	−0.0038 (7)	−0.0022 (6)
C11	0.0262 (9)	0.0210 (8)	0.0227 (8)	−0.0018 (7)	−0.0007 (7)	0.0027 (7)
C12	0.0282 (9)	0.0219 (8)	0.0166 (8)	0.0012 (8)	−0.0005 (6)	0.0014 (6)
C13	0.0296 (10)	0.0295 (9)	0.0206 (8)	−0.0065 (8)	−0.0028 (7)	−0.0026 (7)
C14	0.0267 (9)	0.0268 (8)	0.0206 (8)	−0.0064 (8)	0.0011 (7)	−0.0005 (7)

### Geometric parameters (Å, °)

**Table:**

O1—C1	1.3506 (19)	C7—N1	1.345 (2)
O1—H1O	0.839 (10)	C8—N2	1.354 (2)
O2—C7	1.237 (2)	C8—C9	1.485 (2)
O3—C8	1.223 (2)	C9—C10	1.392 (2)
O4—C12	1.3497 (19)	C9—C14	1.400 (2)
O4—H4O	0.846 (10)	C10—C11	1.388 (2)
C1—C6	1.392 (2)	C10—H10	0.9500
C1—C2	1.396 (2)	C11—C12	1.398 (2)
C2—C3	1.385 (2)	C11—H11	0.9500
C2—H2	0.9500	C12—C13	1.390 (2)
C3—C4	1.395 (2)	C13—C14	1.385 (2)
C3—H3	0.9500	C13—H13	0.9500
C4—C5	1.393 (2)	C14—H14	0.9500
C4—C7	1.477 (2)	N1—N2	1.3856 (18)
C5—C6	1.386 (2)	N1—H1N	0.877 (10)
C5—H5	0.9500	N2—H2N	0.874 (10)
C6—H6	0.9500		
			
C1—O1—H1O	110.1 (18)	N2—C8—C9	116.72 (15)
C12—O4—H4O	112.4 (19)	C10—C9—C14	118.62 (15)
O1—C1—C6	117.41 (15)	C10—C9—C8	117.85 (15)
O1—C1—C2	122.70 (15)	C14—C9—C8	123.53 (16)
C6—C1—C2	119.88 (15)	C11—C10—C9	121.10 (16)
C3—C2—C1	119.90 (16)	C11—C10—H10	119.5
C3—C2—H2	120.1	C9—C10—H10	119.5
C1—C2—H2	120.1	C10—C11—C12	119.54 (17)
C2—C3—C4	120.52 (16)	C10—C11—H11	120.2
C2—C3—H3	119.7	C12—C11—H11	120.2
C4—C3—H3	119.7	O4—C12—C13	117.63 (15)
C5—C4—C3	119.15 (15)	O4—C12—C11	122.39 (16)
C5—C4—C7	122.84 (15)	C13—C12—C11	119.98 (15)
C3—C4—C7	118.02 (15)	C14—C13—C12	119.96 (16)
C6—C5—C4	120.74 (16)	C14—C13—H13	120.0
C6—C5—H5	119.6	C12—C13—H13	120.0
C4—C5—H5	119.6	C13—C14—C9	120.80 (17)
C5—C6—C1	119.80 (16)	C13—C14—H14	119.6
C5—C6—H6	120.1	C9—C14—H14	119.6
C1—C6—H6	120.1	C7—N1—N2	119.10 (14)
O2—C7—N1	120.37 (14)	C7—N1—H1N	125.3 (15)
O2—C7—C4	122.52 (15)	N2—N1—H1N	114.8 (16)
N1—C7—C4	117.11 (14)	C8—N2—N1	119.49 (14)
O3—C8—N2	120.18 (16)	C8—N2—H2N	121.9 (16)
O3—C8—C9	123.09 (17)	N1—N2—H2N	114.5 (16)
			
O1—C1—C2—C3	178.36 (16)	N2—C8—C9—C14	9.1 (3)
C6—C1—C2—C3	−1.0 (3)	C14—C9—C10—C11	0.4 (3)
C1—C2—C3—C4	−0.4 (3)	C8—C9—C10—C11	−179.92 (17)
C2—C3—C4—C5	1.0 (3)	C9—C10—C11—C12	0.6 (3)
C2—C3—C4—C7	−179.43 (16)	C10—C11—C12—O4	178.43 (16)
C3—C4—C5—C6	−0.2 (3)	C10—C11—C12—C13	−1.1 (3)
C7—C4—C5—C6	−179.74 (16)	O4—C12—C13—C14	−178.89 (17)
C4—C5—C6—C1	−1.2 (3)	C11—C12—C13—C14	0.6 (3)
O1—C1—C6—C5	−177.59 (17)	C12—C13—C14—C9	0.3 (3)
C2—C1—C6—C5	1.8 (3)	C10—C9—C14—C13	−0.9 (3)
C5—C4—C7—O2	152.83 (17)	C8—C9—C14—C13	179.49 (17)
C3—C4—C7—O2	−26.7 (2)	O2—C7—N1—N2	−4.4 (2)
C5—C4—C7—N1	−27.4 (2)	C4—C7—N1—N2	175.75 (14)
C3—C4—C7—N1	153.10 (16)	O3—C8—N2—N1	−7.3 (3)
O3—C8—C9—C10	10.2 (3)	C9—C8—N2—N1	173.48 (15)
N2—C8—C9—C10	−170.57 (16)	C7—N1—N2—C8	−72.5 (2)
O3—C8—C9—C14	−170.11 (19)		

### Hydrogen-bond geometry (Å, °)

**Table:**

*D*—H···*A*	*D*—H	H···*A*	*D*···*A*	*D*—H···*A*
O1—H1o···O2^i^	0.84 (1)	1.85 (1)	2.684 (2)	172 (3)
O4—H4o···O3^ii^	0.85 (1)	1.83 (1)	2.675 (2)	178 (3)
N1—H1n···O2^iii^	0.88 (1)	2.08 (1)	2.920 (2)	162 (2)

Symmetry codes: (i) −*x*+3/2, −*y*+1, *z*+1/2; (ii) −*x*+3/2, −*y*+2, *z*−1/2; (iii) *x*−1/2, −*y*+3/2, −*z*+1.
